# GlycoDash: automated, visually assisted curation of glycoproteomics datasets for large sample numbers

**DOI:** 10.1007/s00216-025-05794-3

**Published:** 2025-02-22

**Authors:** Tamas Pongracz, Steinar Gijze, Agnes L. Hipgrave Ederveen, Rico J. E. Derks, David Falck

**Affiliations:** Center for Proteomics and Metabolomics, Leiden University Medical Center, Leiden, The Netherlands

**Keywords:** Glycoproteomics, Data curation, Glycomics software, Antibody glycosylation, High-throughput glycomics, LC–MS

## Abstract

The challenge of robust and automated glycopeptide quantitation from liquid chromatography-mass spectrometry (LC–MS) data has yet to be adequately addressed by commercial software. Recently, open-source tools like Skyline and LaCyTools have advanced the field of label-free MS^1^ level quantitation. Yet, important steps late in the data processing workflow remain manual. Because manual data curation is time-consuming and error-prone, it presents a bottleneck, especially in an era of emerging high-throughput methodologies and increasingly complex analyses such as antigen-specific antibody glycosylation. We addressed this gap by developing GlycoDash, an R Shiny-based interactive web application designed to democratize label-free high-throughput glycoproteomics data analysis. The software comes in at a stage where analytes have been identified and quantified, but whole measurement and individual analyte signals of insufficient quality for quantitation remain and reduce the quality of the overall dataset. GlycoDash focuses on these challenges by incorporating several options for measurement and metadata linking, spectral and analyte curation, normalization, and repeatability assessment, and additionally includes glycosylation trait calculation, data visualization, and reporting capabilities that adhere to FAIR principles. The performance and versatility of GlycoDash were demonstrated across antibody glycoproteomics data of increasing complexity, ranging from relatively simple monoclonal antibody glycosylation analysis to a clinical cohort with over a thousand measurements. In a matter of hours, these large, diverse, and complex datasets were curated and explored. High-quality datasets with integrated metadata ready for final analysis and visualization were obtained. Critical aspects of the curation strategy underlying GlycoDash are discussed. GlycoDash effectively automates and streamlines the curation of glycopeptide quantitation data, addressing a critical need for high-throughput glycoproteomics data analysis. Its robust performance across diverse datasets and its comprehensive feature toolbox significantly enhance both research and clinical applications in glycoproteomics.

**Graphical Abstract:**

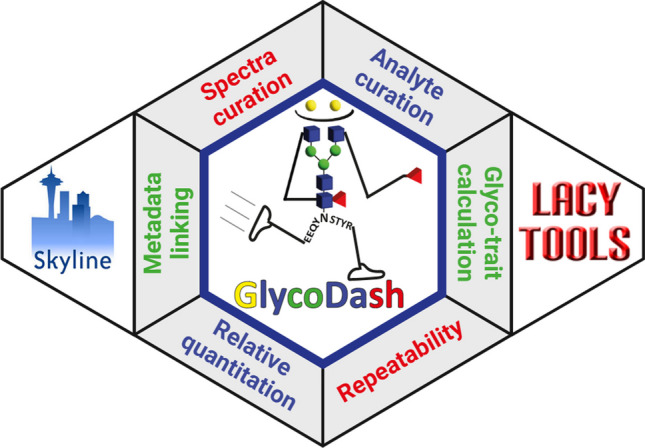

**Supplementary Information:**

The online version contains supplementary material available at 10.1007/s00216-025-05794-3.

## Introduction

Glycosylation is a non-template-based post-translational modification that profoundly impacts protein stability and function [1, 2]. Most eukaryotic proteins undergo extensive site-specific carbohydrate modifications as they traverse the secretory pathway [3]. These highly heterogeneous carbohydrates are collectively known as glycans. Alterations to the glycan moiety have been observed in response to numerous diseases, leading to the demand and subsequent rise of techniques allowing sensitive profiling and relative quantification of protein glycosylation at scale [4–7].

Liquid chromatography (LC)-mass spectrometry (MS)-based technologies are extensively used for glycosylation profiling and benefit from improvements in sample preparation, separation, and innovative data acquisition strategies that enable clinical cohort-scale high-throughput analyses involving thousands of samples [5, 8, 9]. Some of these approaches rely on the enzymatic or chemical liberation of glycans from their carrier proteins, sacrificing protein- and site-specific information. Conversely, bottom-up glycoproteomics retains site-specificity: this glycopeptide-centric approach employs proteases that cleave the proteins into a mixture of peptides and glycopeptides. Here, the peptide backbone serves as a barcode, representing its source protein and glycosylation site(s), which is essential to profile glycosylation microheterogeneity [10]. Relative quantitation is usually based on label-free quantitation of the full scan parent spectrum, but targeted approaches have also been reported [8, 11, 12].

Despite attracting considerable attention for its broad spanning physiological roles and dysregulation in most diseases, large-scale protein glycosylation analysis using label-free bottom-up glycoproteomics data is still challenging*.* Initial identification of glycopeptides can be performed automatically with search engines [13, 14], but manual checks or refinements are often necessary, especially to assign the correct isomer. Skyline [15] and LaCyTools [16] are open-source software packages commonly used for (pre-)processing of (high-throughput) LC–MS glycopeptide data. These popular tools can perform retention time alignment, spectral calibration (except Skyline), analyte quantitation, and calculation of quality metrics. Before a final dataset can be (statistically) analyzed and visualized, it needs to be curated, removing spectra and analytes of insufficient quality or incorrect assignments. This has hitherto been a largely manual process, lacking dedicated computational tools.

To address this gap, we developed GlycoDash, an R Shiny-based web application featuring an intuitive, interactive graphical user interface designed for automated, visually assisted curation of large glycoproteomics datasets. GlycoDash starts at a stage in the data processing and analysis workflow where glycopeptides have been identified and quantified by other tools, but whole measurements and individual analytes of insufficient quality for quantitation limit the quality of the dataset. GlycoDash intends to solve practical issues by offering several robust options to streamline glycoproteomic data analysis, such as linking measurement-associated information and metadata, spectral and analyte curation, and normalization, but it does not aid glycan structure identification. Additionally, it provides various optional features accessible with a single click, including repeatability metrics summaries, built-in and customizable glycosylation trait calculations, absolute quantification, and data exploration tools. Regarding the optional features, such as visualization and exploration, various tools are available, for example, Glynsight and other Expasy resources [17, 18]. Finally, GlycoDash enables users to download the curated data at every analysis step, to generate R Markdown-based HTML reports documenting curation settings and associated visualizations, and to obtain a curated, machine-readable output ready for statistical analysis.

## Experimental section

### Software and hardware

GlycoDash is an open-source R Shiny [19] dashboard that is available under an MIT license (https://github.com/Center-for-Proteomics-and-Metabolomics/glycodash), and was developed within the Golem [20] framework, promoting an organized, maintainable, and scalable approach for building interactive web applications. We provide an extensive user’s guide on GitHub which provides a step-by-step protocol on the use of GlycoDash, as well as additional explanations and screenshots of the features. We recommend installing the Docker container, which includes the correct R version and all dependencies (see Supplementary Methods, section “Installation and requirements”), and will use 4 gigabytes (GB) of disk space. For individual use, a standard desktop or laptop computer is sufficient, though 16 GB of random-access memory (RAM) is recommended. GlycoDash was developed and tested on a laptop containing a 1.3 gigahertz (GHz) Intel® Core™ i5-1235U CPU and 16 GB of RAM. We performed calculations within the Docker container running on a server—8 GB of RAM, 2.40 GHz Intel® Xeon® E5-4657L v2 CPU—which allowed fast data processing by at least five users without noticeable interruptions.

### Datasets and samples

For the purpose of the description and evaluation of GlycoDash, the following publicly available LC–MS datasets were re-used.

#### Dataset 1

A total of 1581 measurements of IgG1 Fc *N*-glycosylation from plasma samples of the BEAT-COVID cohort, 734 targeting anti-SARS-CoV-2 spike protein (anti-S) IgG1 and 847 targeting total plasma IgG1, were re-used from an article by Pongracz et al. [21]. BEAT-COVID is a single-center cohort study including longitudinal plasma samples of 159 polymerase chain reaction (PCR)-confirmed SARS-CoV-2 patients infected during the first and second pandemic waves. Pooled plasma from healthy volunteers (VisuCon), negative for the antibodies against the SARS-CoV-2 spike protein (anti-S IgG), was used as negative controls. Positive controls were prepared by pooling small aliquots of patient plasma.

#### Dataset 2

A dataset of total serum IgG, IgA, and IgM *N*- and *O*-glycosylation in a subset of the Pregnancy-induced Amelioration of Rheumatoid Arthritis (PARA) cohort was re-used from van Tol et al. [22]. The data is available via the ProteomeXchange accession PXD057528.

#### Dataset 3

Furthermore, we re-used a small dataset on glycoengineered anti-human immunodeficiency virus (HIV) monoclonal antibodies (mAbs) recently published and available via the ProteomeXchange accession PXD053363 [23].

#### Dataset 4

To exemplify GlycoDash on Skyline data and demonstrate the treatment of isomeric glycans/glycoconjugates, we used a publicly available glycomics dataset (ProteomeXchange accession PXD029644) of 24 *O*-glycosylation measurements of keratinocyte cell lines with various knock-outs in the glycosylation pathway [24]. A total of 49 *O*-glycan structures had been targeted with Skyline, including several isomeric ones.

### General glycosylation analysis

GlycoDash was developed for data from glycopeptide-centered LC–MS approaches. In this manuscript, we exemplify the software with several applications in the field of antibody glycosylation. In general, antibodies of interest are purified via affinity interactions with either a specific antigen or anti-human isotype-specific antibody fragments. After proteolytic cleavage, glycopeptides are separated by nano-reversed phase-LC and measured by nano electrospray ionization-time of flight-mass spectrometry. Semi-automatic quantification by LaCyTools is based on the MS^1^ sum spectra of the elution times of interest [16]. Separation is dominated by the peptide backbone, especially when using an ion pairing reagent. Otherwise, increased hydrophobicity of the protonated carboxylic acid group leads to additional separation by the number of sialic acids. More details on the method are available as a published protocol [25]. The method details for the BEAT-COVID dataset, the monoclonal anti-HIV antibodies, and serum/plasma IgG, A, and M analysis are further described in the source manuscripts [21–23].

We also analyzed glycan-centric LC–MS data, specifically a cell-line *O*-glycosylation dataset featuring chemically liberated *O*-glycans measured by C18 nanoLC-MS [24]. Liberation under basic, non-reducing conditions was followed by hydrazide enrichment, 2-aminobenzamide labeling, and cotton hydrophilic interaction chromatography–solid-phase extraction. Integration of LC-MS^1^ extracted ion currents was performed with Skyline [15].

### Considerations for quantitation software output formatting

Skyline output needs to contain at least three unique columns, which are “Protein name,” “Peptide,” and “Precursor Charge,” and three outputs, which are “Isotope Dot Product,” “Average mass error PPM,” and “Total area MS1.” “Protein name” should indicate the glycosylation site for glycoconjugate data, and we used “AB” here to signify released glycan data. “Peptide” and “Precursor Charge” contained the glycan name in the same naming convention as typically employed by LaCyTools users (see below) and the charge state as a number, respectively. Other glycan names are allowed for the “Peptide” column, but then glycosylation trait calculations must be customized. The “Isotope Dot Product” is a measure of similarity to the theoretical isotopologue pattern expected for the given elemental composition with 1 being a perfect match. Samples are structured column-wise, meaning each sample gives a column for each of the three outputs.

In LaCyTools, the syntax “peptide1H5N4F1S1” is used for analyte names (glycopeptide with five hexoses (H), four *N*-acetylhexosamines (N), one fucose (F), and one *N*-acetylneuraminic acid (S)) which is determined in the analyte reference file. “Analyte Intensity” (background subtracted) and “Analyte QC” have to be selected in the “Output Format” tab of LaCyTools. This will automatically create all necessary output for further processing in GlycoDash. Note that the “isotopic pattern quality” (IPQ) output of LaCyTools measures the deviation of the observed isotopologue pattern from the theoretical pattern. In contrast to the “isotope dot product” output by Skyline, where a higher number indicates a better match, a score of 0 for the IPQ indicates a perfect match.

### Data structure, import, and metadata merging

In the “Data Import” tab (see Supplementary Methods, section “User interface overview”), a LaCyTools summary*.txt* file or a Skyline output*.csv* file was uploaded. Sample IDs were linked to measurement file names containing the plate number and position, e.g., “plate1_C10,” using an Excel file with the layout currently optimized for 96-well plates. Sample types were automatically extracted from the sample IDs. GlycoDash presents these to the user who can (1) accept them or (2) reject, go back, and correct the sample names or (3) provide a list matching sample types and sample IDs. Glycosylation sites were also automatically detected. In LaCyTools data, the first part of the analyte name, ending before the first number, indicates the glycosylation site, e.g., in IgGI1H4N4F1, “IgGI” would denote the conserved Fc glycosylation site of IgG1. In Skyline, the glycosylation site would be extracted from the “Protein Name” column. Optionally, metadata can be uploaded and linked to the imported data. Matching of metadata and measurement data is automatically performed by matching the same sample IDs in both inputs and combining the respective rows from both files into one row. Thus, a sample ID column should be included in the metadata file.

### Spectra curation

The following cutoffs for the quality criteria (see Supplementary Methods, section “Spectra curation”) were selected for LaCyTools data—mass accuracy − 20 to + 20 ppm, isotopic pattern quality 0.2, and signal-to-noise 9—and Skyline data—mass accuracy − 2 to + 2 ppm, isotopic dot product 0.9, and total area 0. Spectra were curated per glycosylation site based on their sum intensity and on the percentage of analytes passing all three quality criteria cutoffs. The BEAT-COVID and the Skyline dataset were curated with the negative control option (95th percentile), using anti-S-negative pooled healthy donor plasma (VisuCon) and a fetuin standard as negative controls, respectively. For the IgG/A/M dataset, the lowest second percentile of all samples (excluding controls) was removed. For anti-HIV mAbs data, spectra curation was skipped. All measurements (individual glycosylation sites) falling below the passing analyte percentage and/or sum intensity cutoffs were excluded by GlycoDash from further processing steps and no values were reported.

When using negative controls for spectra curation, GlycoDash determined the cutoffs as the highest values of passing analyte percentage and spectrum sum intensity found in the controls. However, a percentile value of included controls was set (default 95%) that may exclude the most extreme values to limit the impact of potential outliers. Alternatively, in the absence of relevant negative controls, GlycoDash calculated the cutoffs in such a way that a user-defined percentile of spectra with the lowest values for total spectrum intensity and percentage of passing analytes were excluded. The percentile is applied to each individual cutoff dimension. Therefore, the percentage value of spectra excluded will be slightly higher than the percentile value.

### Analyte curation

In the processing step following spectra curation, thus using only the remaining measurements, GlycoDash checked for each analyte the relative frequency of samples in which it passes the quality criteria (Supplementary Methods, section “Analyte curation”). In contrast, the anti-HIV mAbs and the cell lines were evaluated individually “per sample.” For the BEAT-COVID dataset, the “per biological group” option was chosen, distinguishing between ICU and non-ICU patients, and using only anti-S IgG measurements (thus excluding total IgG measurement and applying the result to these samples). For the IgG/A/M dataset, the “on all data” option was used. The frequency cutoff was set to 50% in both cases. Values for analytes that do not pass the quality criteria were removed by GlycoDash, either “per sample” individually or from all measurements where a consensus list is generated either from the whole dataset or by inclusive merging of the biological groups’ lists. After applying the analyte curation, GlycoDash normalized the data per glycosylation site (total area normalization) and visualized the outcome as a heatmap-like plot (Figs. S3 and S5) either comparing glycosylation sites or samples.

### Glycosylation trait calculations

GlycoDash uses predefined formulae with fixed compositions, instead of a logic-based categorization of compositions. The Supplementary Methods file, section “Glycosylation traits,” provides a detailed description. In version 1.6.5, automated glycosylation trait calculations are available for human IgG, IgA, and IgM *N*-glycans, human IgA *O*-glycans, human joining chain *N*-glycans, and murine IgG *N*-glycans. Compositions that are not present in the dataset are ignored. If novel compositions are included in the dataset, a warning message appears detailing the compositions that could not be processed. The user can then download the incomplete formulae, add their novel compositions, and use the custom glycosylation traits option. For other scenarios, the custom glycosylation trait option can be used with an entirely newly created list. In a Microsoft Excel file, the formula is specified according to the following syntax. Two columns are headed with “trait” and “formula” in the first row. The “trait” column is filled with the names of the glycosylation traits, the only restriction being the exclusion of spaces. To the “formula” column, the row-wise matched calculations of the traits are added. The full analyte names, including both peptide identifier and glycan composition, are used and separated from other analytes and mathematical operators by spaces. Three modifications are typically used: (1) summing multiple analytes into a trait using “ + ” (2) modifying relative intensities of individual analytes by a correction factor—for example, “0.5 *” for monogalactosylated, diantennary glycans when calculating modification per antenna, and (3) renormalization to a subset by division (“/”) through the sum of that subset—such as fucosylation of complex type glycans.

### Optional features used

For the BEAT-COVID dataset, optional features were used. IgG glycosylation traits were automatically calculated in GlycoDash. Technical variability was derived from repeated measurements of a pool of patient samples. Additional insights on a per batch/plate level can be gained from the HTML report. For example, mean and coefficient of variation (CV) were plotted per batch/plate, and median inter- and intra-plate CV were calculated.

## Results and discussion

**Fig. 1:**
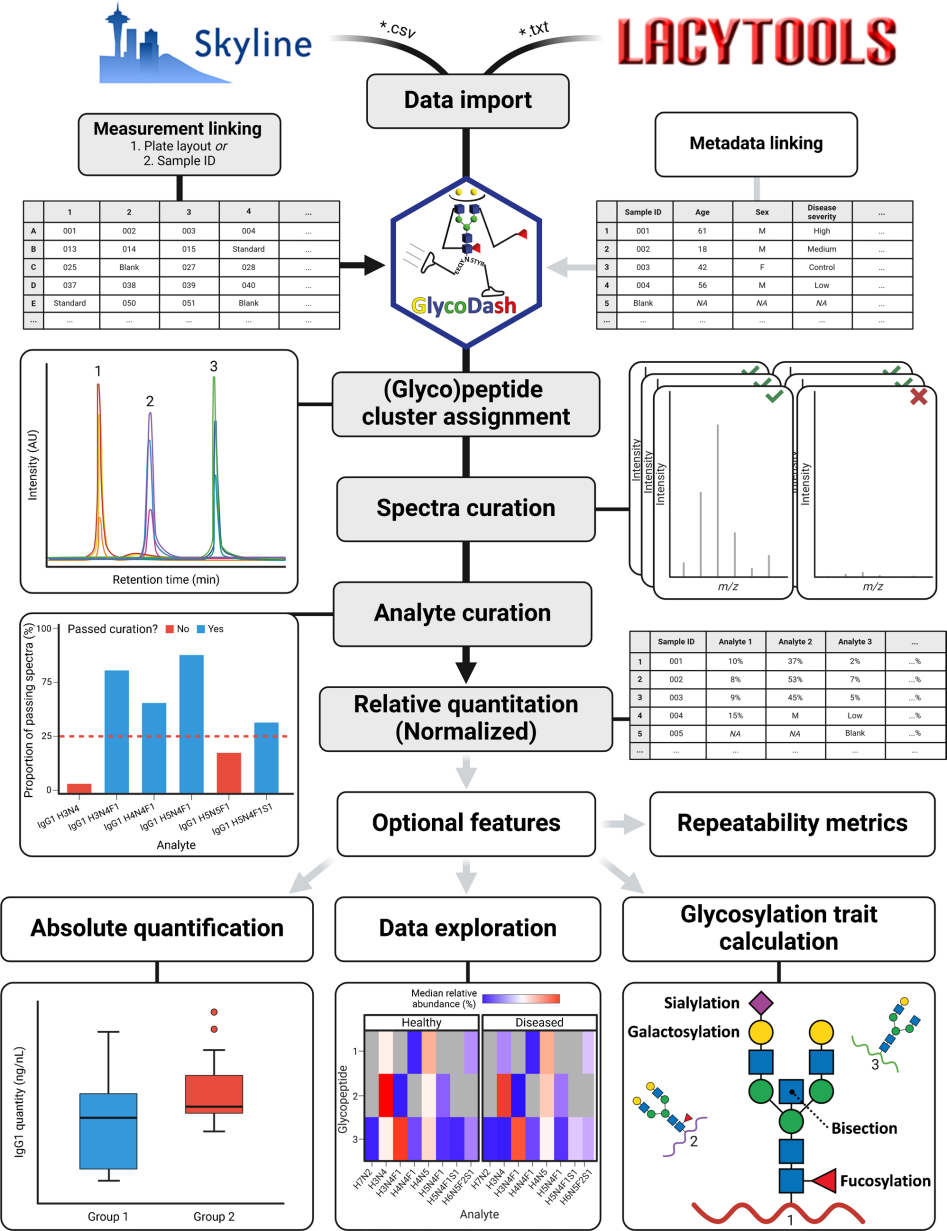
Schematic overview of the steps and features automated through GlycoDash, with mandatory steps and optional features depicted in gray and white, respectively

GlycoDash was developed to facilitate the quantitative comparison of LC–MS glycoproteomics data, addressing the need to curate these datasets and integrate biological or clinical metadata. An intuitively structured dashboard provides a low engagement hurdle and immediate responsiveness. Curation is needed to remove measurements of insufficient quality (spectral curation) and to decide which analytes can be measured at sufficient quality in the present dataset (analyte curation), thus improving the overall quality and reliability of the final processed dataset. Ultimately, the user receives an output ready for statistical analysis and final visualization. GlycoDash achieves this aim through the stepwise process outlined in Fig. 1 which is supplemented by several optional features that help the user explore their data and perform additional calculations. An extensive user’s guide with practical explanations of the individual processing steps is available at (https://github.com/Center-for-Proteomics-and-Metabolomics/glycodash). Glycoproteomics data, sample identities, and metadata are provided as separate files. GlycoDash merges all input files and extracts several important qualifiers. For example, it groups glycopeptides per corresponding glycosylation sites and samples to sample types. GlycoDash requires quantities of glycans, glycopeptides, or other glycoconjugates, as well as three associated analyte quality measures: mass error, isotopologue pattern quality, and signal-to-noise (or signal intensity). In the current version (1.6.5), GlycoDash can automatically process output from Skyline [15] and LaCyTools [16]. The “Considerations for quantitation software output formatting” of the “Experimental section” describes the program settings necessary to obtain a GlycoDash-compatible output format.

We welcome suggestions to integrate output from alternative programs as long as they contain the required (or comparable) values, are well-maintained, and have a robust user base. Import/merging is followed by spectral curation which is based on sum intensity of all analytes and fraction of quantifiable analytes. In the next step, a consensus list of analytes is created based on the fraction of samples in which a specific analyte can be reliably quantified and by taking into account potential qualitative differences between experimental and clinical groups (see analyte curation using biological groups).

Finally, total area normalization is applied to calculate relative glycoform abundances per glycosylation site. The relative glycoform abundances, and optionally glycosylation traits, with associated metadata can be exported for further analysis and visualization. An interactive, time-stamped HTML report with all processing settings and visualizations is made available, for convenient recording of selected curation parameters and visualizations, and for easy sharing of results, to facilitate adherence to FAIR principles. In this context, we recommend providing the HTML report as supplementary information with publications.

### User interface

**Fig. 2:**
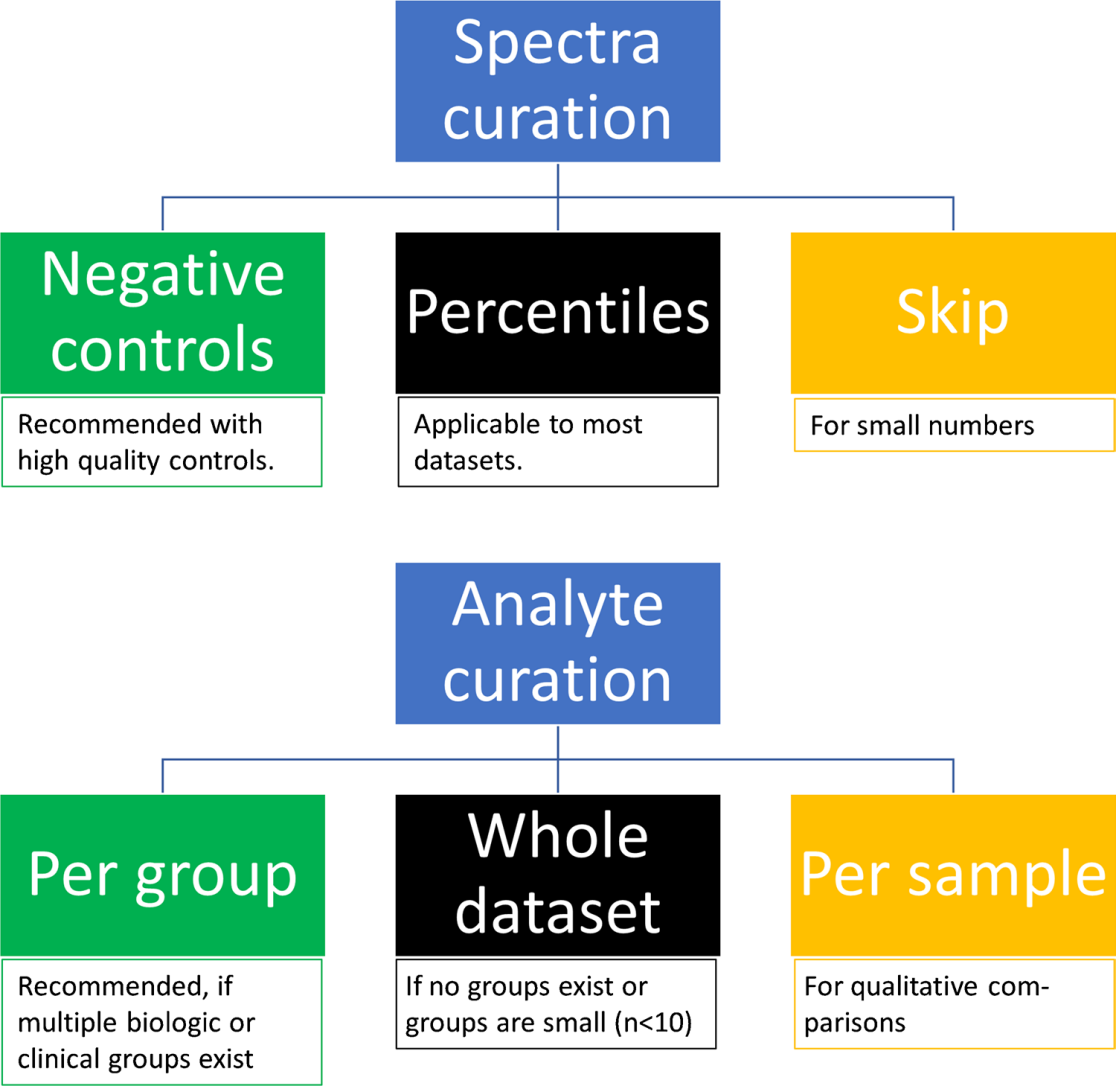
Overview and decision tree for spectra curation and analyte curation core features

The user interface is a browser-based dashboard, separated into various tabs which contain the individual steps of the processing/analysis (see Supplementary Methods, section “User interface overview”). User input and commands are collected via buttons, checkboxes, text/number fields, dropdown menus, and sliders, as appropriate. Non-obvious steps requiring user action are accompanied by a tooltip with additional information (mouseover info icon), a compromise between providing said information and keeping the user interface tidy and accessible. In addition, examples can be downloaded via paper clip icons, demonstrating the syntax of required input documents. While performing calculations, the program will display a loading screen indicating that the program is still active. Outcomes of calculations will be visualized per glycosylation site in secondary tabs within the respective processing step tab where each secondary tab may show several panels when groups are distinguished in curation (Fig. S1). All visualizations are interactive with the possibility to zoom-in, exclude grouping variables, where applicable (e.g., sample types), or mouseover for more information on individual samples (e.g., sample ID). Each processing step, following data import, offers the possibility to export the data in its current state and to circumvent the step, facilitating a modular use of individual GlycoDash functions (Fig. 2).

### Curation of measurements

**Fig. 3:**
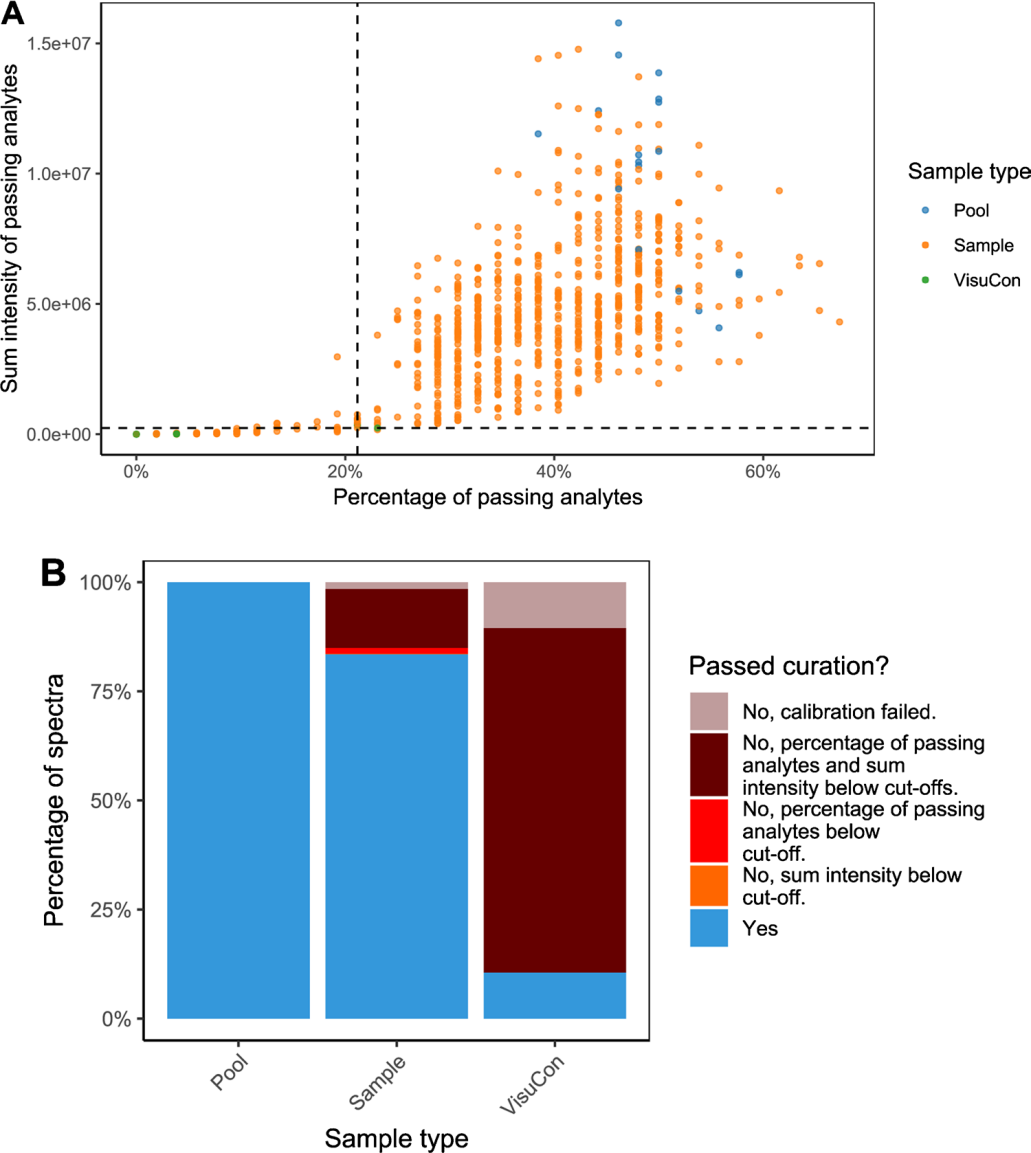
Spectra curation outcome visualization for anti-S IgG1 in the BEAT-COVID dataset. **A** Distribution and cutoffs of spectrum quality. The colors indicate different sample types which can be individually (de-)selected (Fig. S2) to focus on specific types, e.g., controls. **B** Overview of excluded measurements per sample type. Reasons for exclusion are color coded and, in GlycoDash, mouseover provides numbers and percentages of in-/excluded measurements per group. Extensive tables with curation results per sample or analyte are also available

The bases of both curation steps are the three imported quality parameters. The mass error and the agreement of the isotopologue distribution with the theoretically calculated counterpart (isotopologue pattern quality value for LaCyTools and isotope dot product for Skyline) of each analyte confirm that the targeted elemental composition is the dominant signal within the integration boundaries. The signal-to-noise ratio (LaCyTools) or the absolute signal (Skyline) are used to exclude analytes of insufficient abundance to be reliably quantified. If a glycopeptide is present in multiple charge states, they will be curated individually. The user sets boundaries for the quality parameters or uses the default, empirically defined values (see Methods section “Spectra curation”), for sufficiently similar measurement methodologies [25]. GlycoDash determines for every analyte in each sample whether all quality parameters fall within the boundaries. For each individual glycosylation site in each sample, the program divides the number of analytes that passed this check by the total number of analytes in the targeted extraction list. GlycoDash also calculates the sum intensity of all analytes that passed the aforementioned criteria. These two values are then displayed per sample in a scatter plot (Fig. 3).

The user can choose from three alternatives regarding spectral curation, but all are performed separately per glycosylation site. (1) The user may skip spectral curation, for example, if the dataset is small and each sample unmissable for data analysis (see anti-HIV mAbs dataset). Note that the GlycoDash spectral curation is often a second-pass measurement curation as measurements of obviously poor quality likely have been removed during alignment and calibration steps in LaCyTools or by manual checks in Skyline. (2) The recommended method is to base spectral curation on highly relevant negative control measurements, but these are rarely trivial to obtain. Controls in the same matrix are preferred over buffer blanks in bioanalysis as they give a complete picture of the background signal. Sometimes negative controls might not yield values, in which case the outcome of this curation is identical to not applying curation. (3) Therefore, in the absence of adequate negative controls or values for them, the user may choose to exclude a fixed portion of the lowest quality measurements to improve the overall data quality. For this option, it is important to exclude all types of controls and blanks for which GlycoDash provides a dropdown menu in the respective text box (see Supplementary Methods, section “Spectra curation cut-offs”). Regardless of whether curation is based on negative controls or a fixed portion, non-normal distribution is assumed, and thus, the user selects a percentile of either the negative controls or the considered dataset at which the cutoff is set. Alternatively, an advanced setting for the negative control option allows the assumption of normal distribution and cutoffs based on mean and variance of the negative controls. Based on the selected option and settings, GlycoDash calculates a cutoff value for total spectrum intensity and percentage of passing analytes per glycosylation site, and visualizes the cutoffs in a scatter plot together with the distribution of samples (Fig. 3). Interactivity of this plot allows to focus on certain regions of the distribution (through zooming), certain sample types (through colors and simple exclusion) and outliers (mouseover provides sample name, sample ID, and values). Figure 3 shows the results of the spectra curation corresponding to measurements of glycopeptides from a single glycosylation site, namely the conserved Fc *N*-glycosylation site of SARS-CoV-2 spike protein-specific (anti-S) IgG1 (EEQYNSTYR), in the BEAT-COVID dataset. The bulk of the measurements forms a dense cluster, but those that were excluded form an obviously separate “tail” to the lower left corner, meaning that they are consistently poor in both considered quality dimensions. Note that in this scenario, poor measurement quality can have technical reasons but likely rather results from low specific antibody concentrations in certain samples. The negative control option (95th percentile) was used for this dataset, employing an anti-S-negative pooled healthy donor plasma (VisuCon) as negative control. Measurements which did not have values for a given glycosylation site (spectra failing calibration in LaCyTools) were excluded from this calculation (default; alternative option to set these to zero available). Reassuringly, all buffer blanks were below the cutoff (Fig. S2) and positive controls (pool) all passed spectral curation (Fig. 3). Similar settings were used and similar outcomes were observed in the Skyline dataset (Fig. S3A). Spectra curation is performed independently per glycosylation site, customizing curation to the site, as data quality often varies significantly between them. This is exemplified by the IgG/A/M glycosylation dataset where the IgG1 and IgA N144/131 glycosylation sites generally showed high-quality spectra, IgM N209 spectra of lower quality, and IgA N47 the poorest quality spectra. Consequently, three (IgG1 and IgA N144/131), four (IgM N209), and seven (IgA N47) samples were excluded from the analysis of the specific glycosylation site (Figure S1).

### Analyte curation and selection of a consensus list

**Fig. 4:**
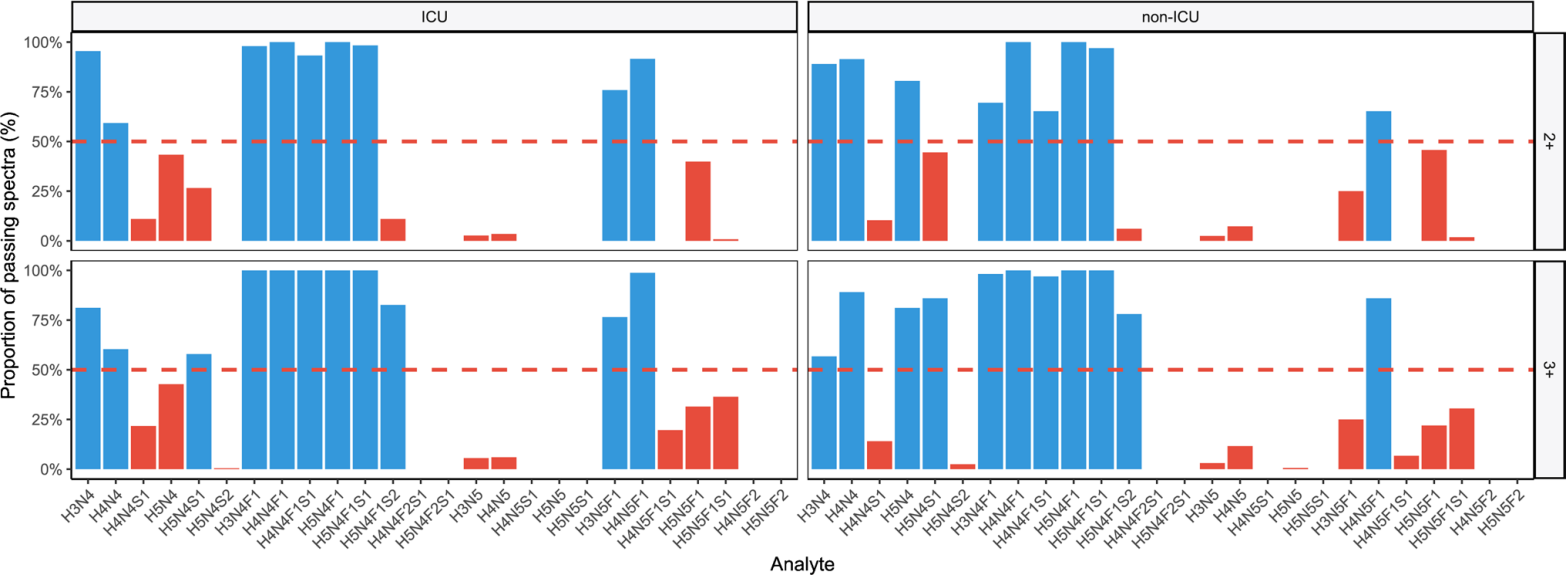
Visualization of the analyte curation step exemplified on the anti-S IgG1 BEAT-COVID data. Bars represent the relative frequency with which each charge state of an analyte fulfills the three quality criteria. The red dotted line visualizes the user-chosen minimum frequency for inclusion, with blue and red bars belonging to analyte charge states included in or excluded from the analyte list (per biological group; here ICU and non-ICU)

Once the dataset has been reduced to measurements of acceptable quality, the next step decides which analytes will be quantified. This is based on the previous check whether an analyte in a specific sample meets all quality parameters, but now results are visualized and evaluated per analyte. Three scenarios are distinguished:

1. For each measurement, the unique results of the previous check are saved (to assess measurements individually);
2. An analyte list is compiled directly from the whole dataset (Fig. S4);
3. For each biological group, an analyte list is compiled separately which is merged into a consensus list using AND/OR-logic and the consensus list is applied to the whole dataset (Fig. 4).

For options 2 and 3, one or several bar graphs, respectively, are generated showing the relative frequency with which the analyte quality check is passed per analyte and charge state. Analyte curation per charge state is needed because the quality of charge states may vary significantly between analytes due to size and acidity differences, and specific interferences. The user defines the minimum frequency with which analytes should have sufficient quality. Typical values are 80% for high-quality datasets or 50% for medium- to low-quality datasets when using option 3. We recommend option 3 as it is superior at preserving important qualitative differences between biological groups.

Biological groups can be interpreted as sets of measurements between which glycosylation is expected to differ significantly and thus often derive directly from the scientific hypothesis. In line with statistical analysis, groups should be sufficiently large for curation with an absolute minimum of ten samples. An example of analyte curation for the BEAT-COVID dataset in Fig. 4 distinguished two biological groups. COVID-19 patients, who were admitted to the intensive care unit (ICU), were expected to show differences in anti-S IgG1 glycosylation compared to patients under regular hospital admission or ambulatory treatment (non-ICU). Ultimately, ICU patients showed lower galactosylation and sialylation, and higher bisection and fucosylation at hospital admission [21]. In line with these differences, the anti-S IgG1 compositions H3N5F1 and H5N4 were uniquely curated in the ICU and non-ICU groups, respectively. These two analytes represented the differences between the analyte lists of the two groups and were, according to the AND/OR-logic, incorporated into the consensus list together with all shared analytes. All analytes not on the consensus list were removed from the dataset for all samples.

Sometimes, for example, when the study is highly explorative, there is no basis for distinguishing (large enough) biological groups, in which case option 2 should rather be applied. A lower minimum frequency is often applied to cover more variation within the dataset. When choosing a value below 50%, a prudent manual check of low-frequency analytes is recommended, to manage the impact of potential interferences on the total area normalization. In cases where sample numbers are small, but many experimental, biological, or clinical groups are present, curation per sample can be the best choice (option 1, Figs. S3C and S5). However, we recommend restricting oneself to mainly qualitative comparisons after choosing this option, or, in the absence of (large) qualitative differences, to base additional quantitative comparisons on a curation using option 2.

GlycoDash is equipped to handle data containing isomers. For Skyline data, glycan compositions appearing multiple times on the same glycosylation site are assumed to be isomers and are automatically renamed adding a running lowercase letter (Fig. S3). Note that in LaCyTools, duplicate composition will lead to integration errors. Skyline users can additionally choose to provide unique names for isomers in the Skyline output*.csv* file. It is essential that all isomers are consistently quantified and that retention order is conserved in all samples—both reasonable analytical quality demands for a given dataset—because running letters are assigned in order of appearance. The isomers of the N1 and the H1N1 compositions in Fig. S3C demonstrate that the same isomer is consistently assigned to the same running letter.

Sometimes, it may be desirable to make small adaptations to the curation results. For example, additional identification experiments or manual checks identify an analyte or individual charge state as a false positive. Border cases, like the H5N4 composition in the ICU group or the 2 + charge state of H5N4S1 in Fig. 4, are included to avoid interdependencies between glycosylation traits or improve closeness to the true value at the expense of precision, respectively. Note that in the shown example, H5N4 was automatically included anyway when choosing option 3, as it was passing curation in the non-ICU group. To allow and track such choices, a table with checkboxes and the automated results is available in GlycoDash (Fig. S6). Checkboxes are prefilled with the automated results.

### Glycosylation trait calculation

Glycosylation traits efficiently summarize shared biosynthetic features and reduce the dimensions of the data, thereby allowing easier interpretation of changes in regulation and impact on biomolecular interactions as well as reducing multiple testing burden [5]. Automatic calculation of glycosylation traits in GlycoDash uses predefined formulae with fixed compositions, instead of a logic-based categorization of compositions. Currently, glycosylation traits are directly available via checkboxes for human IgG, A, and M *N*-glycans; human IgA1 *O*-glycans; human joining chain *N*-glycans; and murine IgG *N*-glycans. For other proteins, an Excel file with the formulae can be provided.

### Output, report, and metadata

After performing the analyte curation, the data is automatically normalized to the sum signal area of all analytes included in the consensus list. At this point, the curated data, neatly compiled with associated metadata, can be downloaded as an Excel file or R object. However, we recommend first calculating glycosylation traits [5]. The data is arranged in wide format with columns per metadata, glycosylation trait, and glycan value, row-wise for each sample. Final results are always accessible via the “Export results” tab. There, the interactive R Markdown HTML report can also be downloaded. This report lists the GlycoDash version number, all imported files, all curation setting and the formulas for glycosylation trait calculations. It also contains all visual results of the curation steps and any additional visualizations performed. The report thus enables precise replication of the data curation and detailed inspection of the choices and outcomes at any time in the future.

A recently implemented feature of GlycoDash is the possibility to calculate protein quantities based on spiking with stable isotope labeled (SIL) standards. This was demonstrated using a SIL monoclonal antibody for anti-S IgG1 quantitation from plasma of vaccinees [26]. If several (glyco-)peptide signals are used for quantitation of the same protein, quantitation is based on the median result and the correlation between the different peptides is plotted as a quality check.

### Data visualization

**Fig. 5:**
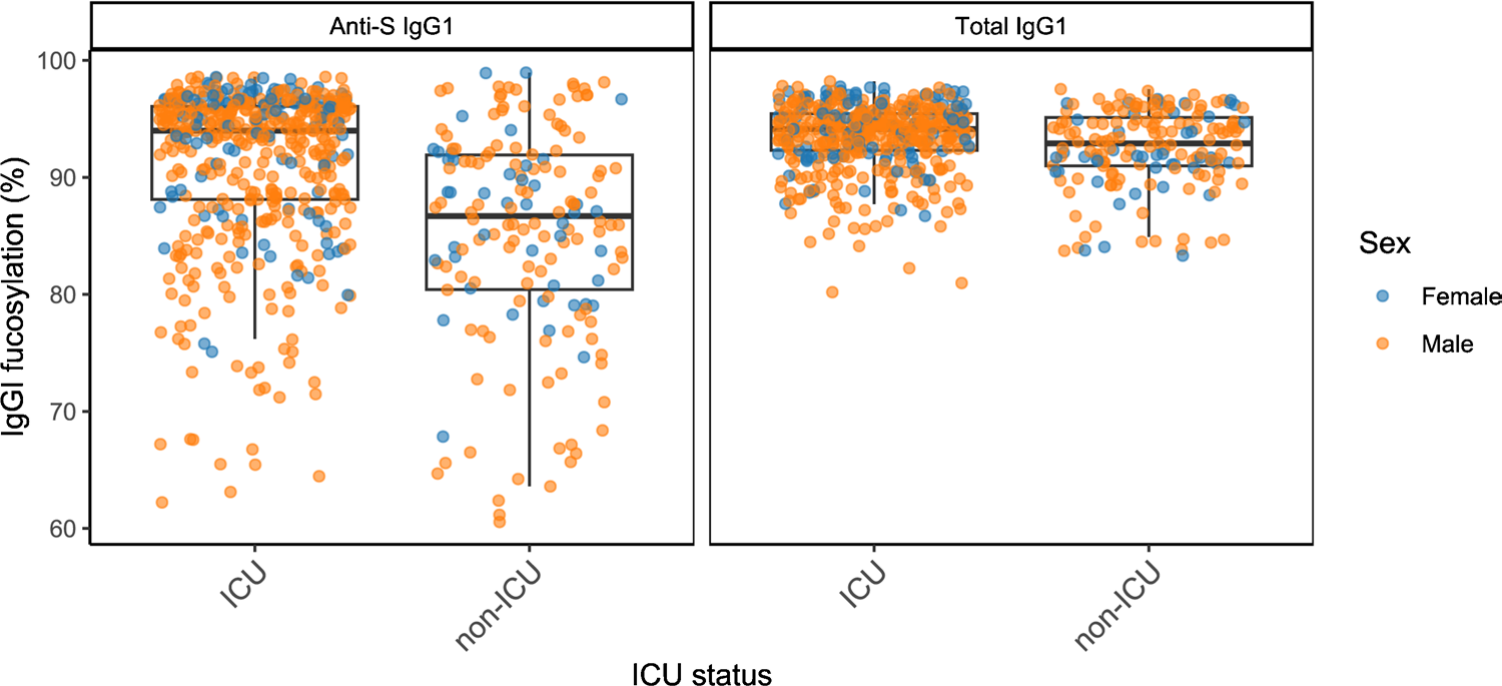
Example of the visualization options for initial data exploration. IgG1 fucosylation is plotted in boxplots comparing ICU and non-ICU patients in the BEAT-COVID dataset, and additionally distinguishing female and male patients. The left panel shows the IgG1 fucosylation of specific anti-S IgG1, and the right panel shows the total plasma antibody repertoire, with the *y*-axis giving a percentage

GlycoDash contains several possibilities for an initial visualization of the curated data, either to perform additional quality checks of the final dataset or to visually explore the data. Under the “Repeatability” tab, glycosylation profiles and their variability can be assessed for technical controls, providing the user with additional indicators of the quality of the dataset. An example of repeated measurements of a positive control pool in the BEAT-COVID dataset can be seen in Fig. S7. The “Data Exploration” tab offers visualization in boxplots, scatter plots, and histograms where any variable from the results file can be used as *x*- or *y*-axis and which can be finetuned, for example, by the exclusion of controls or through coloring by a third variable (Fig. 5).

### Performance

For the exemplified datasets and similar challenges, all individual calculations are performed by GlycoDash in a few seconds (see Methods for hardware requirements). This creates an almost seamless experience that allows the user to finish the curation in a single session which typically last 30 to 60 min, if the user is familiar with GlycoDash and the type of dataset. The seamless performance allows exploration of the impact of different types of settings, for example, when a preferred approach does not generate useful results for a given dataset. GlycoDash enables a useful exploration of dataset types unfamiliar to the user. However, curation goals have to be thoroughly considered. The use of vectorized operations (as opposed to row-wise operations) on data frames proved to be a key choice for performance.

Since dedicated software for high-throughput bottom-up glycoproteomic data processing has hitherto been lacking, performance benchmarking becomes challenging. In-house data processing was commonly performed as per cohort in Excel. With the aim to harmonize divergent practices, we generated a multi-sheet Excel template capable of handling maximum 12 96-well plates. However, this approach required manual data input and was inherently error prone and laborious. GlycoDash was conceptualized and built upon this depreciated/historic Excel template with the aim to simplify and democratize the curation process, and to allow a swifter, streamlined workflow.

## Conclusions

GlycoDash signifies an important step towards full automation of glycomics and glycoproteomics data processing. It automates the essential tasks of curating an acquired dataset, integrating it with metadata, and providing output in a human- and machine-readable format ready for statistical data analysis and visualization. GlycoDash successfully removed both measurements of insufficient quality and analytes that are absent or otherwise could not be quantified in the datasets we tested. This greatly improved the overall quality of the results. By adapting GlycoDash, we could streamline and standardize data curation in our glycomics and glycoproteomics research. This is supported by an interactive report that traces curation choices and outcomes. Wider adaptation in the field is encouraged by an open-source setup of the program. GlycoDash therefore enables FAIR principles, on the one hand with regard to the program by free, persistent access in a well-known repository and by integration of dependencies in a single Docker container, and on the other hand for the curated dataset by tracking all steps, values, and decisions and simplifying integration of metadata. The automated curation using GlycoDash greatly reduced the hands-on time and errors associated with previously used manual curation. Additionally, it was very fast (under 1 min per step), allowing completion in a single session and even permitting the user to rapidly test the impact of different curation choices. Several optional features allow the user to easily visualize and explore the final dataset, so that they can consider the most suitable visualizations or potentially necessary adaptations to the analysis plan.

Most LaCyTools users already base their data curation on a birds-eye view of the quality criteria provided, so for them adaptation of GlycoDash will be straightforward. In contrast, many Skyline users make use of the visual raw data exploration capabilities of Skyline to manually curate their datasets one sample and analyte at a time. It will be interesting to see if Skyline users testing GlycoDash will also adapt the automated, birds-eye curation strategy as stand-alone or will rather use GlycoDash to guide and prioritize manual curation of their datasets.

## Supplementary Information

Below is the link to the electronic supplementary material.

Supplementary file1 (DOCX 861 KB)

Supplementary file2 (DOCX 264 KB)

Supplementary file3 (7Z 30889 KB)
