# Supplementary material for: GlycoDash: automated, visually assisted curation of glycoproteomics datasets for large sample numbers
Source: Anal Bioanal Chem. 2025 Feb 22;417(10):2003–14. doi: 10.1007/s00216-025-05794-3 (PMC11961463; doi:10.1007/s00216-025-05794-3)
Supplement: Supplementary file 1 — Supplementary file1 (DOCX 861 KB) [file 216_2025_5794_MOESM1_ESM.docx]

**GlycoDash: Automated, visually-assisted curation of glycoproteomics datasets for large sample numbers**

Tamas Pongracz^1^, Steinar Gijze^1^, Agnes L. Hipgrave Ederveen^1^, Rico J.E. Derks^1^ and David Falck^1^*

*corresponding author: [d.falck@lumc.nl](mailto:d.falck@lumc.nl)

^1^ Leiden University Medical Center, Center for Proteomics and Metabolomics, Leiden, The Netherlands

ORCIDs:

Tamas Pongracz 0000-0002-8089-4352

Steinar Gijze 0009-0005-0982-7658

Agnes L. Hipgrave Ederveen 0000-0003-1689-0442

Rico J.E. Derks 0000-0002-8920-7133

David Falck 0000-0003-3908-2376

**Table of contents**

**Figure S1**: Spectra curation of IgG/A/M dataset Page 2 to 4

**Figure S2**: Spectra curation visualization for controls of the BEAT-COVID dataset Page 5

**Figure S3**: Excerpts from the GlycoDash analysis of the Skyline dataset. Page 6 to 7

**Figure S4**: Analyte curation of IgG/A/M Page 8

**Figure S5**: Heatmap-type visualization of the anti-HIV mAb results Page 9

**Figure S6**: Summary table of the analyte curation results Page 10

**Figure S7**: Technical variability in the BEAT-COVID-dataset Page 11

**References:**  Page 12

**A

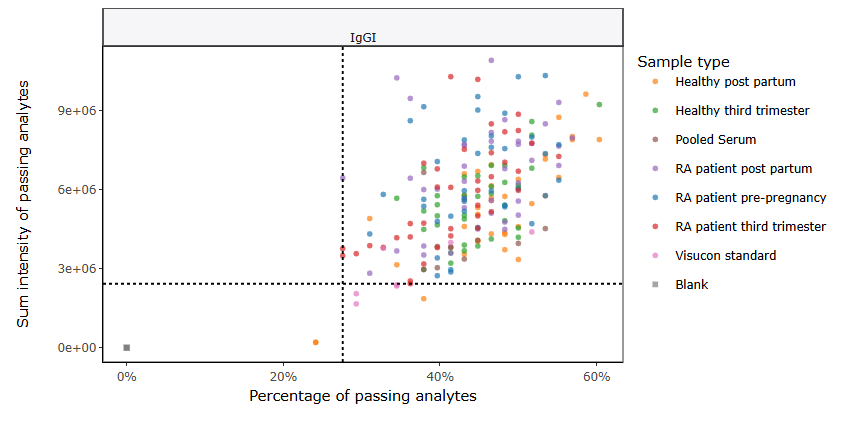
**

**B

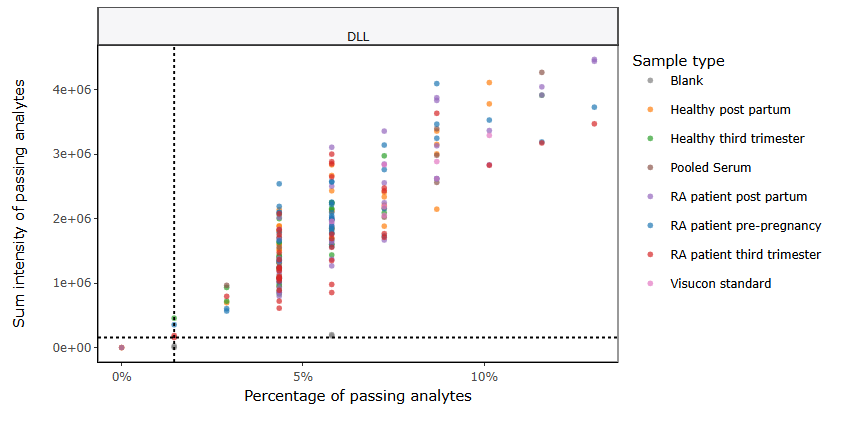
**

**C

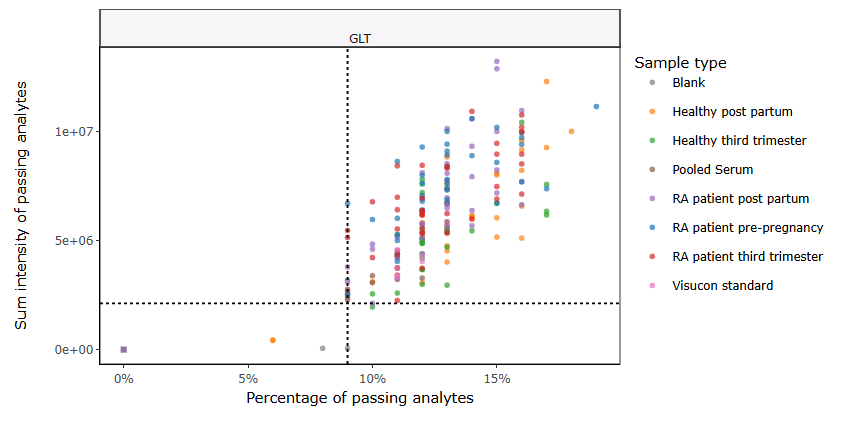
**

**D

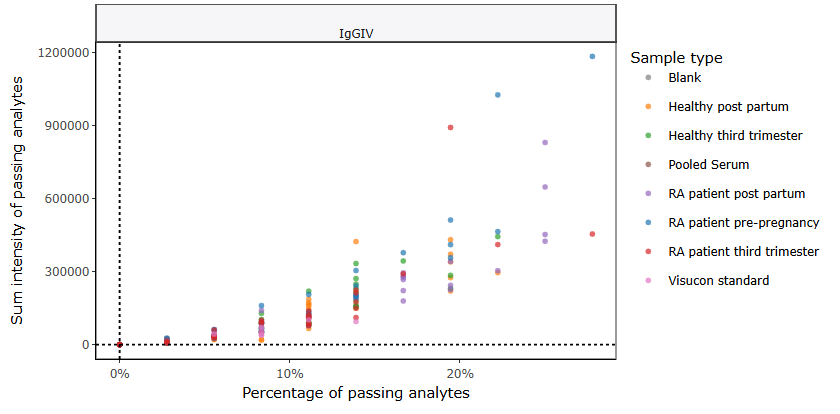
**

**E**

**
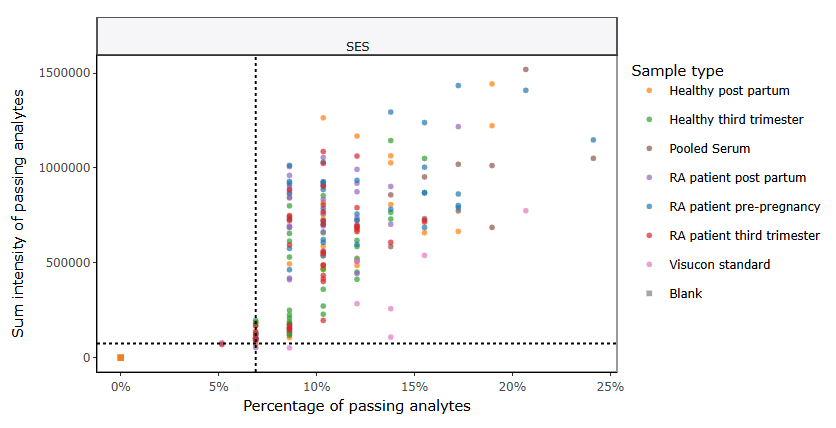
**

**Figure S1**: Spectra curation outcomes from a dataset combining glycosylation analysis of IgG, IgA and IgM (Dataset 2; see **Methods**).^1^ **A)** conserved IgG1 Fc *N*-glycosylation site N297, **B)** shared IgA1/2 *N*-glycosylation site N133/141, **C)** IgM glycosylation site N209, **D)** conserved IgG4 Fc *N*-glycosylation site N297, and **E)** IgA2 C_H_1 *N*-glycosylation site N47.


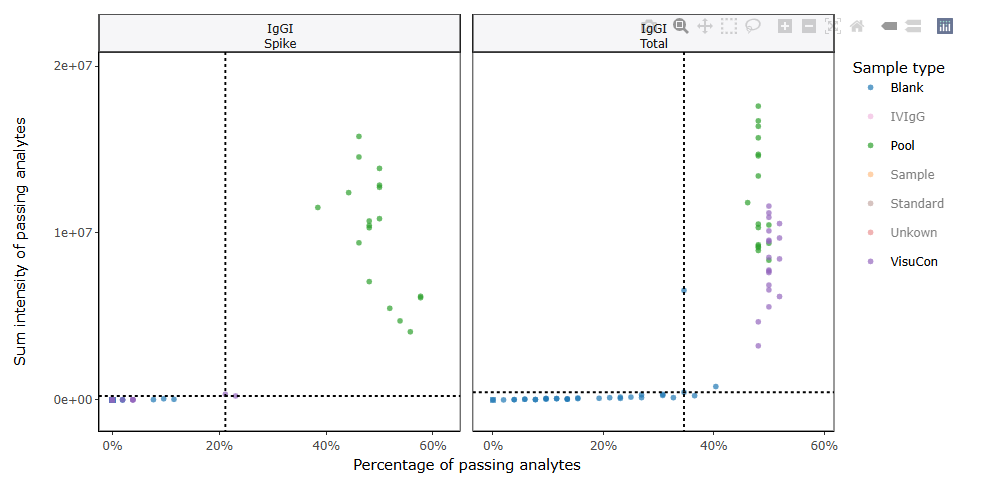


**Figure S2**: Demonstration of the sample type selection feature, visualizing only positive (patient plasma pool) and negative controls (VisuCon) of the BEAT-COVID dataset. In comparison with the spectra curation outcome of the BEAT-COVID dataset shown in **Figure 2**, samples here were de-selected, thus allowing a better view of the outcomes for controls.

**A**


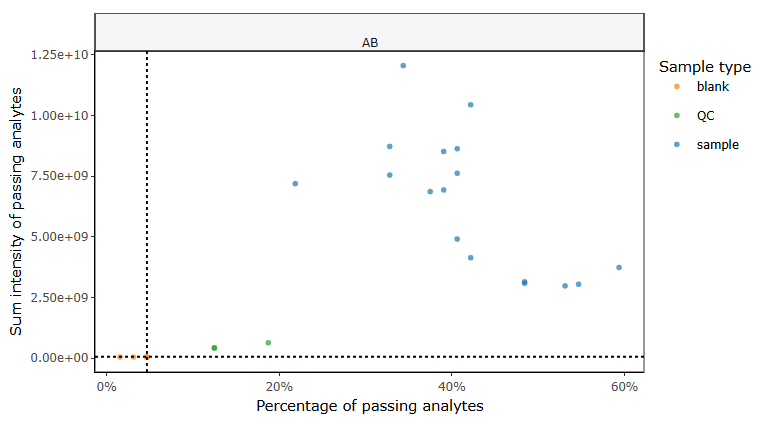


**B**


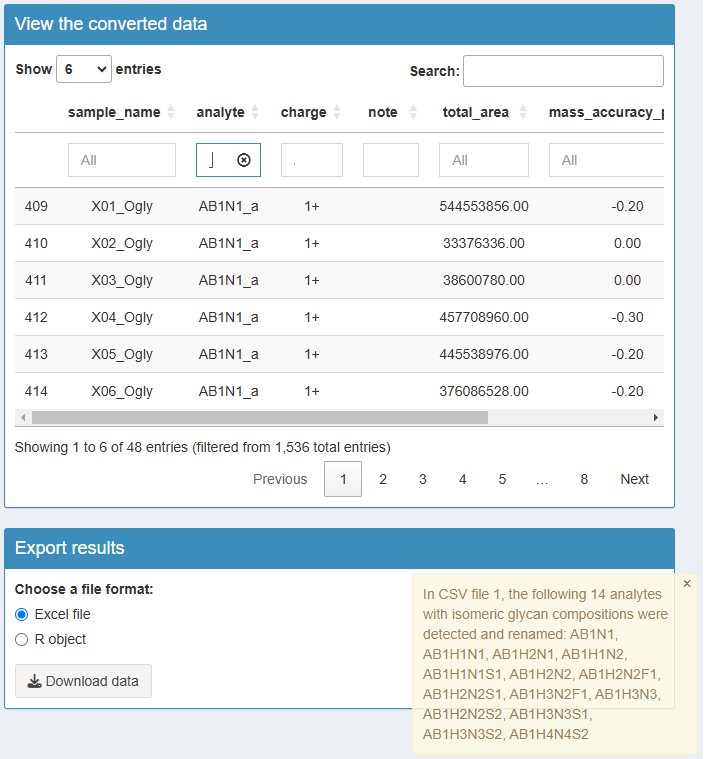


**C

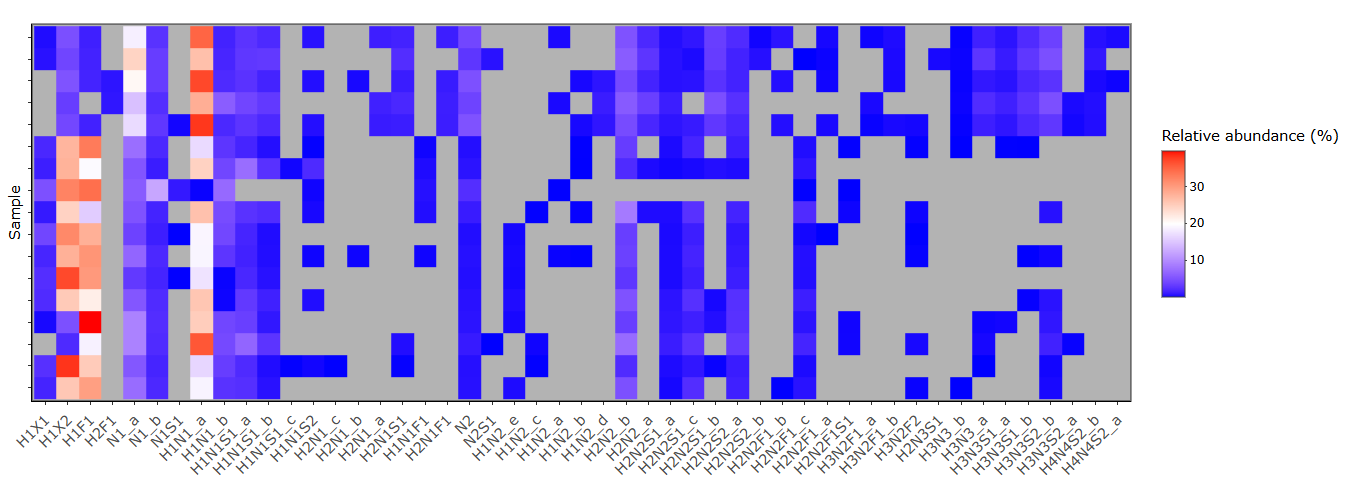
**

**Figure S3**: Excerpts from the GlycoDash analysis of the Skyline dataset of released *O*-glycans in glycoengineered keratinocytes (Dataset 4; **Methods**).^2^ **A)** Spectral curation results, **B)** automated renaming feature, allowing GlycoDash to deal with isomers of identical monosaccharide composition, and **C)** visualization of the final dataset as a heatmap-like plot.

**A

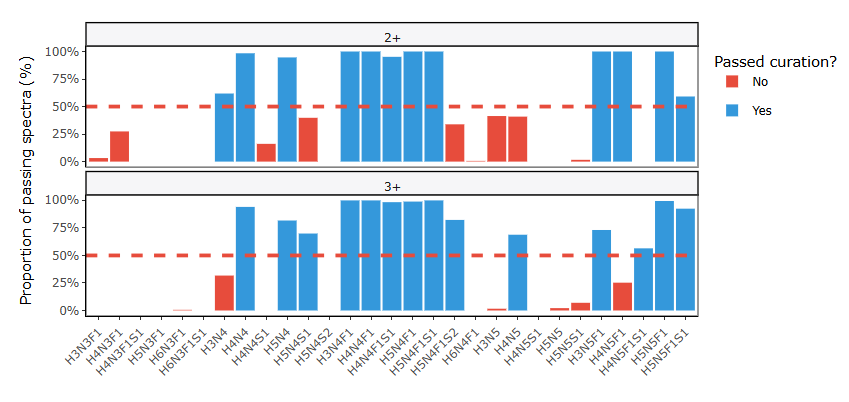
**

**B**

**
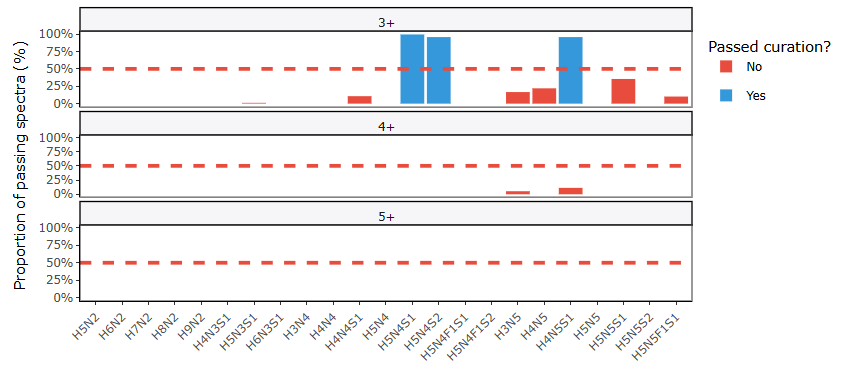

C**
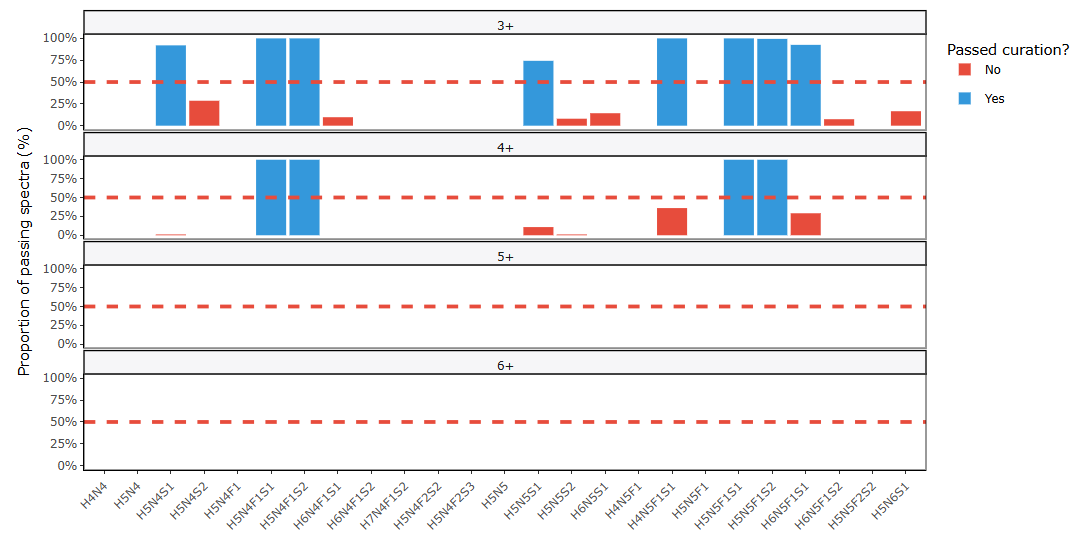


**Figure S4**: Analyte curation outcomes from a dataset combining glycosylation analysis of IgG, IgA and IgM. **A)** Conserved IgG1 Fc *N*-glycosylation site N297. **B)** Shared IgA1/2 *N*-glycosylation site N133/141. **C)** IgM *N*-glycosylation site N209.


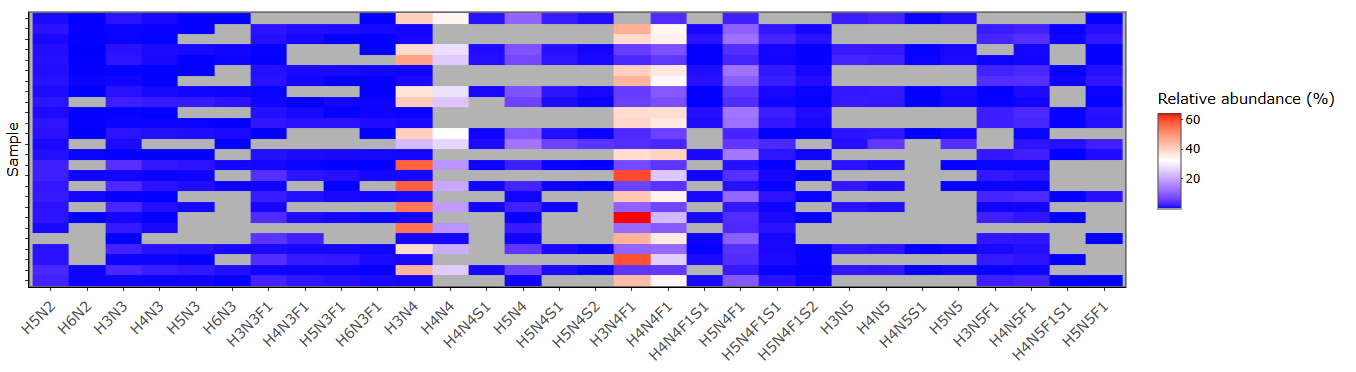


**Figure S5:** Heatmap-type visualization of the anti-HIV mAb results (dataset 3). Each sample has a (potentially) unique analyte list and evaluation should therefore be qualitative in nature. Grey represent analytes that did not pass curation in the respective sample. The analyte lists with major visual differences correspond to wild-type and glycoengineered mAbs. On the major glycans H3N4F1 versus H3N4 and H4N4F1 versus H4N4, the glycoengineering and consequently the fucosylation status are apparent.


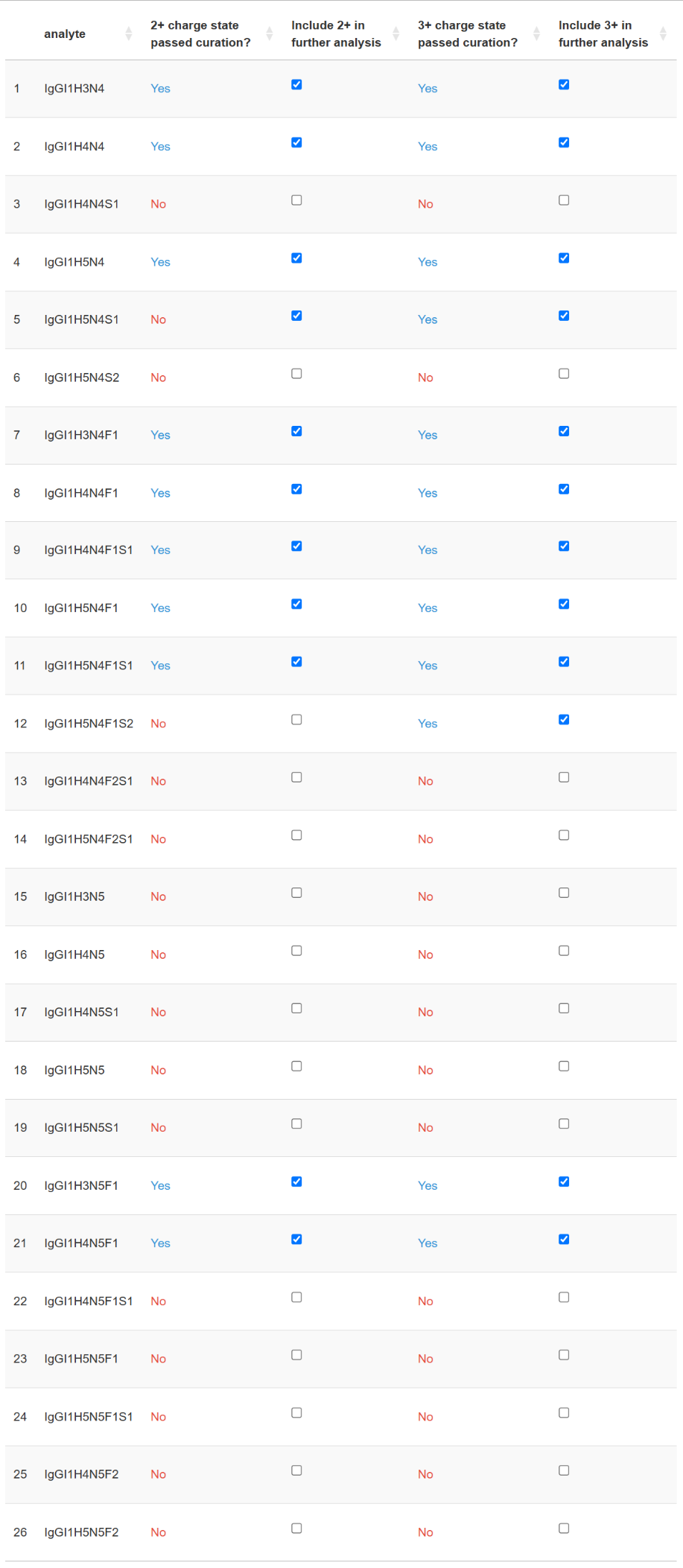


**Figure S6**: Summary table of the analyte curation results. Manual addition or deletion is possible via the tick boxes.


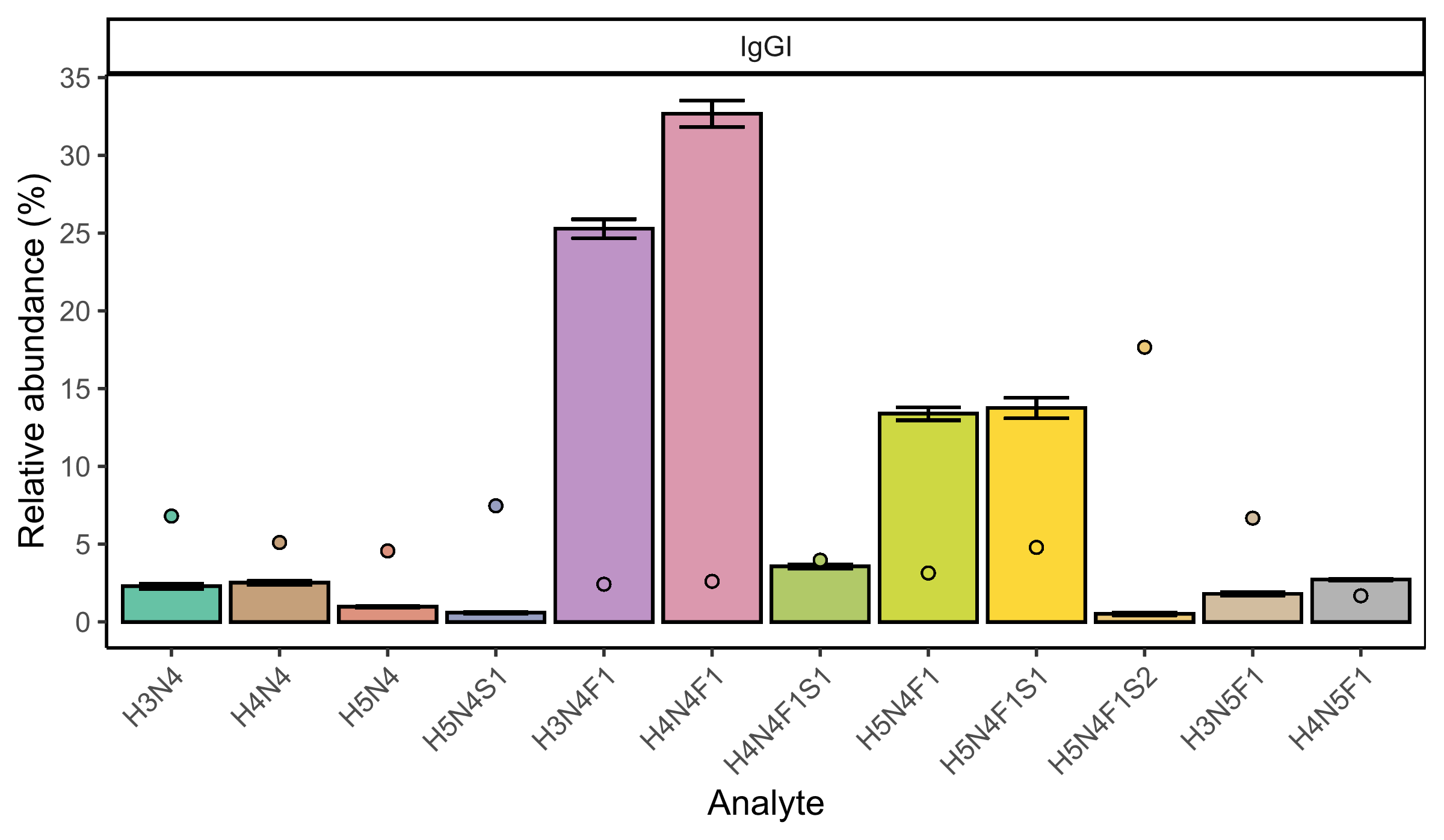


**Figure S7**: Technical variability in the BEAT-COVID-dataset. Means and coefficients of variation (indicated with a circle) of repeated measurements of anti-S IgG1 for a pool of patient samples.

**References**

1 van Tol, B. D. M. *et al.* Comprehensive Immunoglobulin G, A, and M glycopeptide profiling for large-scale biomedical research. *[submitted]* (2024).

2 de Haan, N. *et al.* In-Depth Profiling of O-Glycan Isomers in Human Cells Using C18 Nanoliquid Chromatography-Mass Spectrometry and Glycogenomics. *Anal. Chem.* **94**, 4343-4351 (2022).
