# Supplementary material for: GlycoDash: automated, visually assisted curation of glycoproteomics datasets for large sample numbers
Source: Anal Bioanal Chem. 2025 Feb 22;417(10):2003–14. doi: 10.1007/s00216-025-05794-3 (PMC11961463; doi:10.1007/s00216-025-05794-3)
Supplement: Supplementary file 2 — Supplementary file2 (DOCX 264 KB) [file 216_2025_5794_MOESM2_ESM.docx]

**GlycoDash: Automated, visually-assisted curation of glycoproteomics datasets for large sample numbers**

Tamas Pongracz^1^, Steinar Gijze^1^, Agnes L. Hipgrave Ederveen^1^, Rico J.E. Derks^1^ and David Falck^1^*

*corresponding author: [d.falck@lumc.nl](mailto:d.falck@lumc.nl)

^1^ Leiden University Medical Center, Center for Proteomics and Metabolomics, Leiden, The Netherlands

ORCIDs:

Tamas Pongracz 0000-0002-8089-4352

Steinar Gijze 0009-0005-0982-7658

Agnes L. Hipgrave Ederveen 0000-0003-1689-0442

Rico J.E. Derks 0000-0002-8920-7133

David Falck 0000-0003-3908-2376

**Table of contents**

**Installation and requirements**  Page 2

**User interface overview**  Page 3

**Spectra curation**  Page 4

**Analyte curation**  Page 5 to 6

**Glycosylation traits**  Page 7

**Spectra curation cut-offs**  Page 8

**Installation and requirements**

To install the latest official release of GlycoDash on your computer, follow the steps listed in the README section on GitHub: <https://github.com/Center-for-Proteomics-and-Metabolomics/GlycoDash>

We recommend using Docker to install and run GlycoDash, as this will automatically take care of package and version dependencies (available at https://docker.com/). Alternatively, you can run GlycoDash within RStudio (available at https://posit.co/downloads/). For the development of GlycoDash v1.6.5, R version 4.3.3 is used. The required packages are listed below.

When running GlycoDash in RStudio, having different R and package versions installed may cause GlycoDash to malfunction.

The following R packages are currently used in GlycoDash:

• bsplus (version 0.1.4)

• config (version 0.3.2)

• DT (version 0.33)

• dplyr (version 1.1.4)

• ggplot2 (version 3.5.1)

• golem (version 0.5.1)

• htmltools (version 0.5.8.1)

• kableExtra (version 1.4.0)

• knitr (version 1.49)

• magrittr (version 2.0.3)

• plater (version 1.0.5)

• plotly (version 4.10.4)

• processx (version 3.8.4)

• purrr (version 1.0.2)

• readxl (version 1.4.3)

• rlang (version 1.1.4)

• RLumShiny (version 0.2.3)

• rmarkdown (version 2.29)

• RColorBrewer (version 1.1-3)

• shiny (version 1.9.1)

• shinyalert (version 3.1.0)

• shinyBS (version 0.61.1)

• shinybusy (version 0.3.3)

• shinycssloaders (version 1.1.0)

• shinydashboard (version 0.7.2)

• shinydashboardPlus (version 2.0.5)

• shinyFeedback (version 0.4.0)

• shinyjqui (version 0.4.1)

• shinyjs (version 2.1.0)

• shinyWidgets (version 0.8.7)

• snakecase (version 0.11.1)

• spelling (version 2.3.1)

• testthat (version 3.2.1.1)

• tidyr (version 1.3.1)

• tidyselect (version 1.2.1)

• writexl (version 1.5.1)

**User interface overview**

When launching GlycoDash, you will be presented with the page shown below.


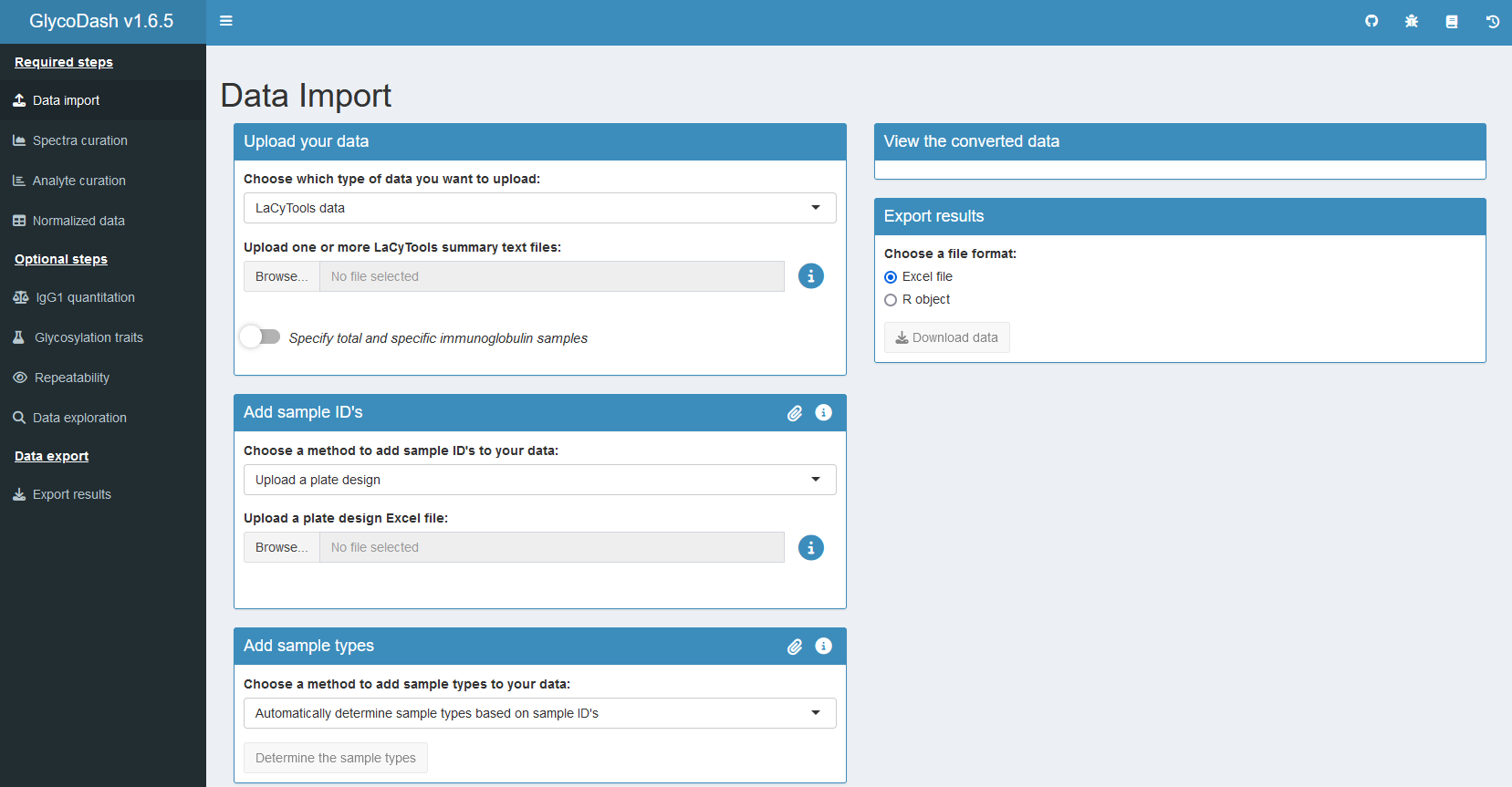


The top-left corner displays the version number of GlycoDash. The left column contains various tabs. The first four tabs (Data Import, Spectra Curation, Analyte Curation and Normalized Data) should be completed in the listed order, as detailed in subsequent sections. Later tabs are optional and can be skipped. The remaining tabs are optional and can be skipped.

The button in the top-right can be clicked to download a changelog of GlycoDash in HTML format.

**Spectra curation**

**Choosing analyte quality criteria**

The analyte quality criteria are used to judge whether the signal of an analyte is of sufficient quality in a given sample to be reliably quantified. It is important to note that different charge states for the same glycopeptide are treated as distinct analytes. This information is used for spectra curation and for the subsequent analyte curation step. Depending on the type of data uploaded, there are three distinct quality criteria for which boundaries can be configured. An analyte is deemed to be of sufficient quality when it meets all three criteria. Only in very specific cases, one or two of the listed quality criteria may be used, by clicking the gears icon and deselecting the criteria that you wish to ignore.

*LaCyTools data:*

- **Acceptable mass accuracy range (ppm)** – Sets the acceptable mass accuracy in ppm. The maximum range is −50 to +50 ppm, with the default being −20 to +20 ppm.
- **Maximum isotopic pattern quality (IPQ) value** – Sets the maximum acceptable IPQ value for an analyte. The better the observed isotopic pattern of an analyte matches its theoretical isotopic pattern, the lower the IPQ will be. The default value is 0.2.
- **Minimum signal-to-noise (S/N) ratio** – The minimum S/N ratio that an analyte should have to be of sufficient quality. The default value is set to 9.

*
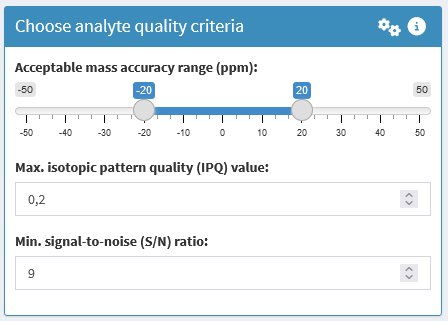
*

*Skyline data:*

- **Acceptable mass accuracy range (ppm)** – Same as for LaCyTools data.
- **Minimum isotope dot product (IDP) value** – The minimum IDP value that an analyte should have. A higher IDP value means that the observed isotopic pattern fits the theoretical isotopic pattern better, with a maximum value of 1. The default value is set to 0.9.
- **Minimum total area** – As Skyline does not output S/N, the total area can be used as an alternative. By default this value is set to 0 (i.e. it is ignored).


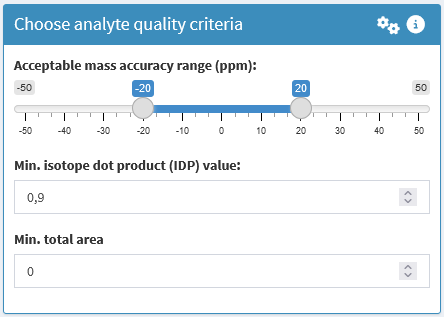


**Analyte curation**

**Analyte curation methods**

There are two main methods for analyte curation. The preferred method is to curate the analytes based on the data.* There are three different ways to do so:

1. **Per biological group** – An analyte passes curation if it meets the quality criteria in a percentage of spectra exceeding the chosen cut-off in at least one biological group (e.g. disease status or treatment). The analyte is then used for total area normalization in all samples, irrespective of the biological group.

- Choose a variable (column) in your data that contains the biological groups.
- Click the “Determine the biological groups” button.
- A popup, displaying detected biological groups, appears for verification.
- Optional: select biological groups to ignore (e.g. when dealing with a small number of samples in a specific group).
- Optional: select sample types to ignore. Sample types without a biological group assigned (e.g. blanks) are automatically excluded, even if not selected here.
- Choose a cut-off or different cut-offs per cluster.
- Click the “Perform analyte curation” button.


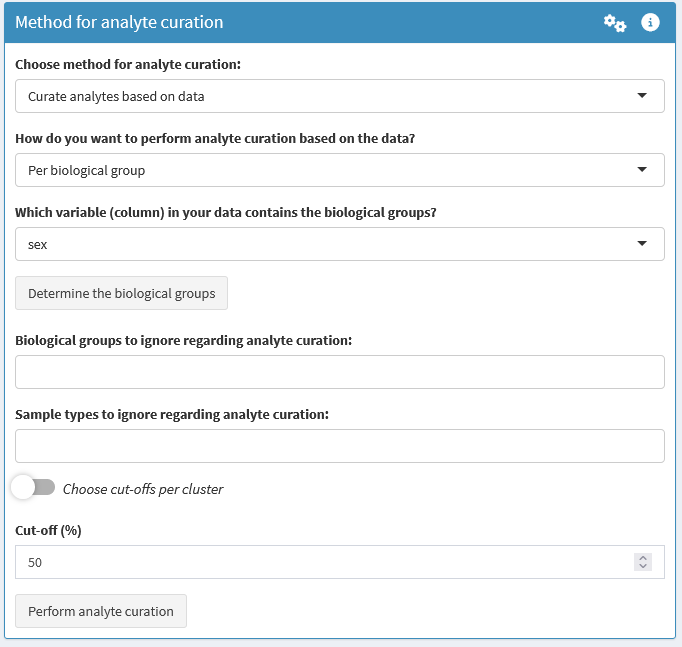


* Alternatively, you can choose to supply an analyte list (as an Excel file or R object) that contains the names of all the analytes that should be kept. All charge states of these analytes will pass curation. This method should only be used if you performed analyte curation based on your data outside of GlycoDash.

1. **On all data** – When an analyte in a given charge state fulfils the quality criteria (as defined in the spectra curation step) in a percentage of spectra exceeding a chosen cut-off percentage, then it passes curation and is used for total area normalization during the next step in all samples.

- There is an option to ignore certain sample types in the assessment. For instance, we recommend to exclude all controls.
- Choose a cut-off, the default is 50%, which is applied to all glycosylation sites. To choose different cut-off percentages per cluster, toggle the “Choose cut-offs per cluster” switch


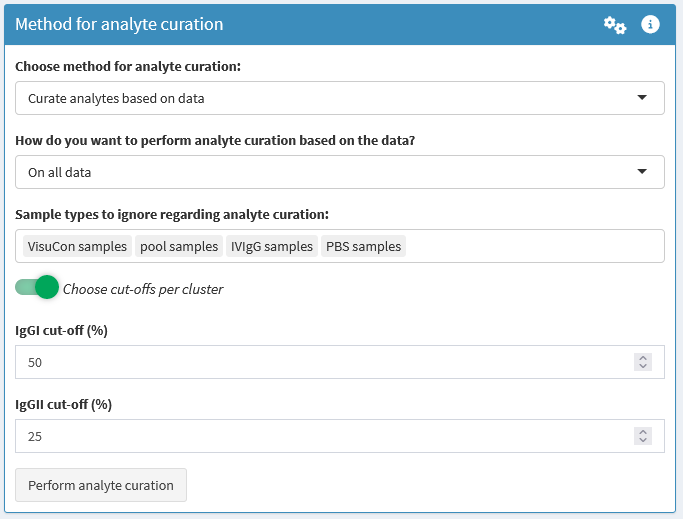


1. **Per sample** – The data is curated per sample. If an analyte meets the quality criteria in one sample, it is used for total area normalization in that sample. However, if the analyte does not meet the quality criteria in another sample, it will not be used for normalization in that particular sample.

**Glycosylation traits**

You can automatically calculate glycosylation traits for human IgG, IgA and IgM (including Joining Chain), and for mouse IgG. These calculations rely on a reference list containing *N*-glycan compositions with known structures, listed in Appendix 2. If your data includes glycan compositions not listed there, a warning message will be displayed when using this option.

1. Select the types of glycans that are present in your data.

2. Choose the traits you wish to calculate.

3. Select the glycosylation sites in your data for which you want to calculate the traits. Then push the “Calculate traits” button.


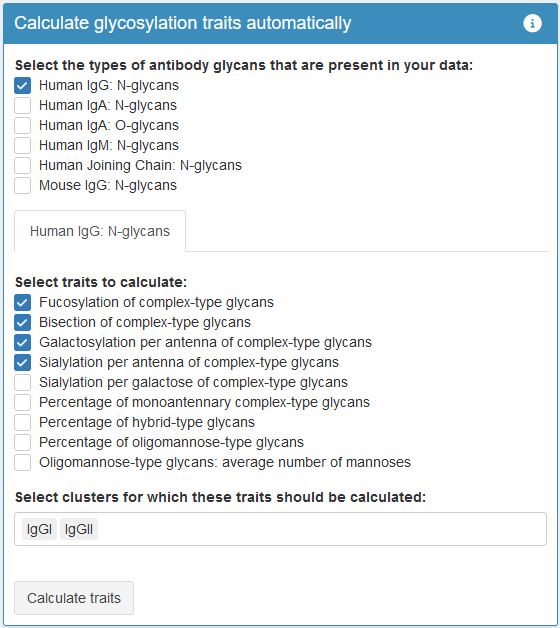


**Spectra curation cut-offs**

For each sample and glycosylation site in your data, the intensities of all passing analytes are summed, and the percentage of passing analytes out of all targeted analytes is calculated. Each spectrum will be curated based on these values. The two available methods for curating the spectra are described below. Alternatively, you have the option to skip spectra curation, in which case all spectra will be utilized in subsequent processing steps.

**Curate spectra based on negative controls:**

1. Choose which sample types should be used as negative controls.
2. Set a percentile of negative control measurements for calculating cut-off values. The default percentile is 95. GlycoDash will automatically compute cut-off values for both the percentage and sum intensity of passing analytes. These cut-offs are calculated separately for each glycosylation site.

- By default, uncalibrated spectra are treated as missing values and are excluded from the calculations. You have the option to include them with a value of zero instead by unchecking the checkbox “Treat uncalibrated spectra as missing values, not zeros”.


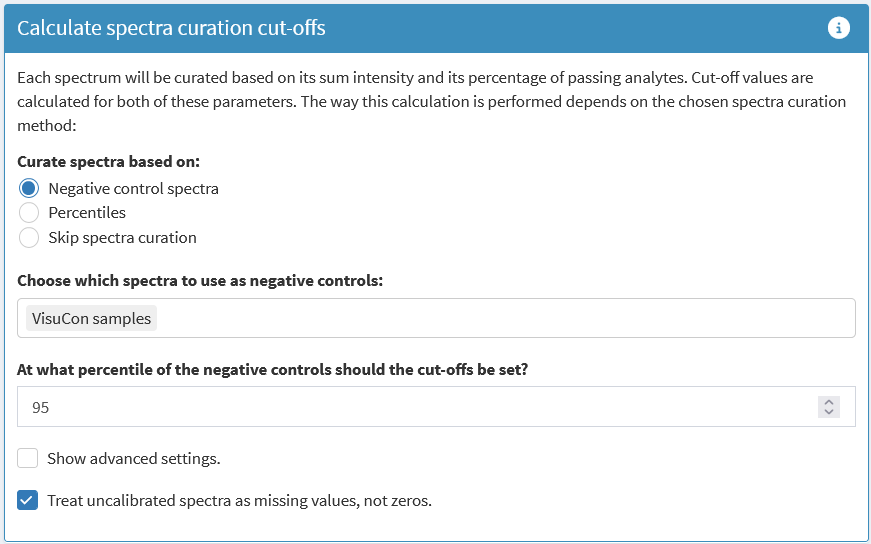


During curation, spectra whose percentage of passing analytes and sum intensity exceed or equal the corresponding cut-off values will pass curation, and will be used for further data processing. In the “Perform spectra curation box”, all spectra are displayed per glycosylation site in an interactive plot, color-coded by sample type. The cut-offs are indicated by dashed bars, and the exact cut-off values are displayed below the plot for reference.
